# Supplementary material for: Spin–Orbit-Induced Nonadiabatic Dynamics: An Exact Ω Representation
Source: J Chem Theory Comput. 2026 Apr 18;22(12):6150–8. doi: 10.1021/acs.jctc.6c00305 (PMC13296488; doi:10.1021/acs.jctc.6c00305)
Supplement: Supplementary file 1 [file ct6c00305_si_001.pdf]

Spin-Orbit Induced Non-Adiabatic Dynamics: An Exact Ω-Representation – supplemental document

Ryan P. Brady* and Sergei N. Yurchenko*

Department of Physics and Astronomy, University College London, Gower Street, WC1E 6BT London, United Kingdom

E-mail: ryan.brady.17@ucl.ac.uk; s.yurchenko@ucl.ac.uk

Functional Forms of the Spectroscopic Model

The $\Lambda - S$ potentials were modeled using simple Morse oscillator functions of the form

$$V(r) = T_e + (A_e - T_e)[1 - \exp(-b(r - r_e))]^2, \tag{S1}$$

where $V(r_e) = T_e$ is the minimum energy, A_e is the dissociation asymptote, b is a parameter controlling the width of the well, and r_e is the minimum position. Using the methodology presented by Brady et al.[1], the SOC is modeled like their diabatic coupling (DC), where combination of two potentials and a NAC define the SOC (DC) via

$$\langle a^3\Sigma^- | \, SO(r) \, | b^1\Sigma^+ \rangle = \frac{1}{2} \tan\left(2\beta(r)\right) (V_b - V_a), \tag{S2}$$

where $\beta_{bX}(r)$ is the mixing angle of the Ω-transformation, defined by the corresponding SO-induced NAC W_{bX} by the cumulative integral.

$$\beta(r) = \beta_0 + \int_{r_0}^{r} W dr. \tag{S3}$$

We model the SO-induced NAC W_{bX} with a Lorentzian function of the form

$$W_{bX}(r) = \frac{1}{2}\frac{\gamma}{\gamma^2 + (r - r_c)^2}, \tag{S4}$$

where γ is the half-width at half-maximum (HWHM) and r_c defines the centroid position which is set to the crossing geometry of the V_X and V_b $(\Lambda - S)$ potentials. The mixing angle β_{bX}, is then

$$\beta_{bX}(r) = \frac{\pi}{4} + \frac{1}{2}\arctan\left(\frac{r - r_c}{\gamma}\right). \tag{S5}$$

The parameters for the potential and NAC curves for this two-state coupled system is given in Table S1.

Table S1: Molecular parameters for the two-state model.

	V_X	V_b	W_{bX}
T_e	0.0000	2000.0000	
A_e	42056.0000	27000.0000	
r_e	1.2100	1.2400	
b	1.9000	2.3000	
r_c			1.4529
γ			0.1000

References

(1) Brady, R. P.; Drury, C.; Yurchenko, S. N.; Tennyson, J. Numerical Equivalence of Diabatic and Adiabatic Representations in Diatomic Molecules. *J. Chem. Theory Comput.* **2024**, *20*, 2127–2139.
